# Status of invasive alien insects of nationwide survey in South Korea

**DOI:** 10.3897/BDJ.12.e133668

**Published:** 2024-10-17

**Authors:** Dayeong Kim, Heejo Lee, Nanghee Kim, Dong Eon Kim

**Affiliations:** 1 Invasive Alien Species Team, National Institute of Ecology, Seocheon 33657, Republic of Korea Invasive Alien Species Team, National Institute of Ecology Seocheon 33657 Republic of Korea; 2 National Ecosystem Survey Team, National Institute of Ecology, Seocheon 33657, Republic of Korea National Ecosystem Survey Team, National Institute of Ecology Seocheon 33657 Republic of Korea; 3 Environmental Impact Assessment Team, National Institute of Ecology, Seocheon 33657, Republic of Korea Environmental Impact Assessment Team, National Institute of Ecology Seocheon 33657 Republic of Korea; 4 Research Policy Planning Team, National Institute of Ecology, Seocheon 33657, Republic of Korea Research Policy Planning Team, National Institute of Ecology Seocheon 33657 Republic of Korea

**Keywords:** nationwide survey, ecosystem-disturbing species, distribution status, natural ecosystems

## Abstract

In this study, we analysed the regional distribution status, species composition differences, seasonal occurrence and habitat types of invasive alien insects that are distirbuted in natural ecosystems nationwide, targeting 3,802 locations in eight regions between 2019 and 2021. A total of 95,017 invasive alien insects belonging to nine orders, 48 families and 77 species were identified. Amongst the taxonomic groups, Hemiptera (35.1%) was dominant, followed by Coleoptera (18.2%) and Lepidoptera (14.3%). Gyeongsangnam-do had the highest percentage of invasive alien insects, with 55 species, while Gyeonggi-do had the highest number of invasive alien insects at 890 sites. We used Non-metric multidimensional scaling (NMDS) to analyse differences in invasive alien insect species composition by region, occurrence by season and habitat type. To compare the differences in invasive alien insect species composition by region, we divided them into four groups (Group 1: Jeollanam-do, Jeollabuk-do; Group 2: Chungcheongnam-do, Chungcheongbuk-do; Group 3: Gyeongsangnam-do, Gyeongsangbuk-do; and Group 4: Gyeonggi-do, Gangwon-do). We found an extensive overlap in invasive alien insects composition by region. Additionally, species composition exhibited seasonal differences, with the highest number of invasive alien insects occurring between July and September. Species occurring in spring (April to May) included *Dryocosmuskuriphilus*, *Hyperapostica* and *Brachyperazoilus*, whereas those occurring in autumn (September to October) included *Vespavelutinanigrithorax*, *Thecodiplosisjaponensis* and *Hermetiaillucens*. Habitat type analysis showed that invasive alien insects lived along roadsides (31.9%), farmlands (27.9%) and parks (19.4%), exhibiting high densities in anthropogenic and disturbed sites, such as parks, residences and farmlands. Ecological statistical analysis showed that the dominance index was 0.6 in Chungcheongbuk-do, the diversity index was 2.7 in Gyeongsangbuk-do, the abundance index was 5.4 in Gyeongsnagnam-do and the equality index was 0.6–0.7 in all regions. Therefore, we aimed to identify the habitat status of introduced and settled invasive insects to provide a basis for selecting primary management target species.

## Introduction

Invasive alien species (IAS) are species whose introduction and/or spread outside their natural habitats threaten biological diversity (Son et al. 2021). With most countries worldwide experiencing economic growth, international trade has also increased, leading to an increase in the introduction of invasive alien species (Vanhanen 2008, Hulme 2009, Meurisse et al. 2019, Hulme 2021). Invasive alien insects can be introduced through the intentional introduction of living organisms for commercial exploitation, biological control, academic purposes or through unintentional introduction, such as mixed with crop seeds or attached to ship's cargo or suitcases (Carrete and Tella 2008, Gertzen et al. 2008, Shimono and Konuma 2008, Bertelsmeier 2021). In particular, insects are likely to spread globally owing to their ease of transportation by hitchhiking in cargo and containers (Hong et al. 2012, Wan and Yang 2016). These invasive alien insects are transported in their egg, larval and adult stages, hatch and repeat their life cycle and settle in new environments (Vanhanen 2008).

Most invasive alien insects in South Korea, including the ant *Anoplolepisgracilipes* Smith F. 1587, *Solenopsisinvicta* Buren 1972 and *Solenopsisgeminata* (Fabricus, 1804) (all Hymenoptera, Formicidae), have been introduced unintentionally in containers and cargo and are often found in or around ports and during container unloading (NIE 2019b, NIE 2020b, NIE 2021b, NIE 2022). No cases of these species being established in South Korea have been reported as of 2020; however, they continue to be introduced and need to be closely monitored. *Linepithemahumile* (Mayr, 1868) (Hymenoptera, Formicidae), which has settled in the Busan Station area was first reported in 2019 (Lee et al. 2020) and *Melanoplusdifferentialis* (Thomas, 1865) (Orthoptera, Acrididae), which has been confirmed in the Onsan Industrial Complex in Ulsan, was first reported in 2018 (Kang et al. 2020). They are thought to have been introduced into the country via cargo and established. These species have been designated as ecosystem-disturbing species because of their potential to disturb or disrupt the ecosystem (Ministry of Environment 2023). In addition, the number of imported seedlings and seeds is increasing due to advances in agricultural technology, the diversification of imported products and markets, the ability to import large quantities of grain through trade between countries and the expansion of floral and horticultural markets (Hulme 2009, Hong et al. 2012). Likewise, the number of greenhouse pests has been found to increase with the increase in the number of imported horticultural products and with the increase in the number of invasive alien insects introduced with nursery stock and seeds (Kiritani 2001). According to Wang et al. (2015), there have been reports of greenhouse pests spreading outdoors, including *Trialeurodesvaporariorum* (Westwood, 1856) (Hemiptera, Aleyrodidae), causing damage and according to Gippet et al. 2019, *Hermetiaillusens* (Linnaeus, 1758) (Diptera, Stratiomyidae) and *Bombusterrestris* (Linnaeus, 1758) (Hymenoptera, Apidae), were introduced for commercial use and then escaped from the facility. In addition native to Southeast Asia and China, these migratory insects are known to cause damage to a variety of crops, including corn and rice (Otuka 2013, Ma et al. 2019, Suh et al. 2021). *Mythimnaseparata* Walker, 1865, *Spodopterafrugiperda* (Smith, 1797) (all Lepidoptera, Noctuidae) and *Nilaparvatalugens* (Stål, 1854) (Hemiptera, Delphacidea) are brought into Korea by spring westerly winds, and are known to travel hundreds to thousands of kilometres (Pedgley 1993, Lee and Uhm 1995, Min et al. 2014, Kim et al. 2018, Lee et al. 2020).

Accurately identifying the timing and means of introduction of invasive alien insects is difficult. Once they become established in natural ecosystems, they can spread rapidly, causing significant ecological and economic damage. Moreover, invasive insects are likely to outcompete native species with similar ecological statuses, causing habitat displacement or population decline, leading to biodiversity loss and negative impacts on agriculture, economy, health and society as a whole (Kenis et al. 2009, Bradshaw et al. 2016). These issues are not unique to South Korea and are becoming a global problem. In 2022, the Convention on Biological Diversity (CBD) announced the Kunming-Montreal Global Biodiversity Framework (GBF) 23 Action Targets. Amongst them, Target 6 aims to identify and manage the pathways of invasive alien species, prevent their introduction and establishment, eliminate and minimise their impacts, eliminate or control their populations and reduce the rate of their introduction and establishment by at least 50% (Convention on Biological Diversity 2023). To achieve these targets, measures are needed to prevent the introduction of invasive alien insects and manage those that have already been introduced.

We surveyed the habitats of invasive insects that have been already introduced and settled in South Korea through various routes. This study analysed the regional distribution status, species composition differences, seasonal occurrence and habitat types of invasive alien insects that are distributed in natural ecosystems nationwide. In the present study, a list of invasive alien insects was established to enable their effective management.

## Materials and methods

### Investigation of invasive alien insects

**Figure 1.**
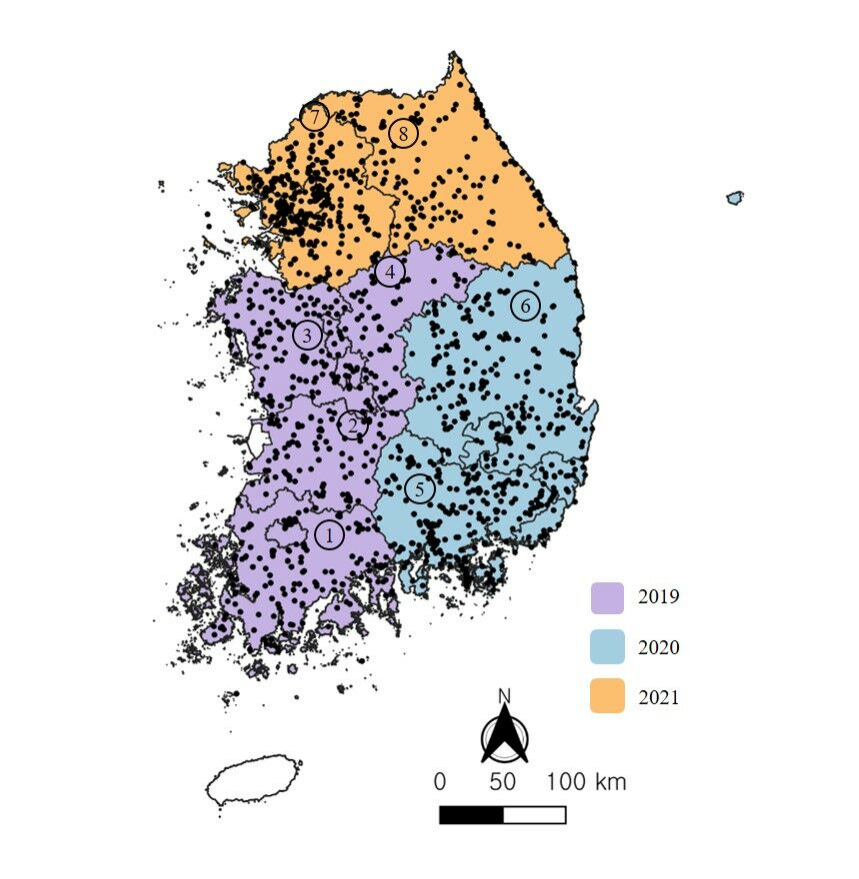
Investigation sites in 2019-2021. 1. Jeollanam-do (with Gwangju Metropolitan City). 2. Jeollabuk-do. 3. Chungcheongnam-do (with Dajeon Metropolitan City and Sejong Special Self-Governing City). 4. Chungcheongbuk-do. 5. Gyeongsangnam-do (with Busan Metropolitan City and Ulan Metropolitan City). 6. Gyeongsanbuk-do (with Daegu Metropolitan City). 7. Gyeonggi-do (with Seoul Metropolitan Government and Incheon Metropolitan City). 8. Gangwon-do.

From 2019 to 2021, surveys of invasive alien insects were conducted in eight regions (159 municipalities) across the country (NIE 2019a, NIE 2020a, NIE 2021a). The surveys were conducted in Jeolla Province (Jeollanam-do, Jeollabuk-do, Gwangju Metropolitan City), Chungcheong Province (Chungcheongnam-do, Chungcheongbuk-do, Daejeon Metropolitan City, Sejong Special Self-Governing Province) in 2019, Gyeongsang Province (Gyeongsangnam-do, Gyeongsangbuk-do, Busan Metropolitan City, Ulsan Metropolitan City, Daegu Metropolitan City) in 2020, Gyeonggi-do (Gyeonggi-do, Seoul Metropolitan City, Incheon Metropolitan City) and Gangwon Province in 2021 (Fig. 1). The coordinates of the survey points were plotted on a map using QGIS (v.3.36.3).

The survey was conducted at least three times per season between March and October (1 hour at each site between 10 a.m. and 6 p.m.), considering habitat types and terrain characteristics. The number of sites by region were as follows: Jeollanam-do (309 sites), Jeollabuk-do (211 sites), Chungcheongnam-do (420 sites), Chungcheongbuk-do (262 sites), Gyeongsangnam-do (745 sites), Gyeongsangbuk-do (628 sites), Gyeonggi-do (890 sites) and Gangwon-do (337 sites). The habitat environment of invasive alien insects was also identified. They were found in environments including grasslands, orchards, roadsides, residential areas, parks, forests and reservoirs and their habitat preferences were analysed.

The survey method was mainly based on visual inspection, depending on the characteristics of each taxon and the surrounding environment of the survey sites. Additional surveys were conducted using tools; brandishing, sweeping and beating were used to catch flying or grass-attached insects using an insect net. An aspirator was used to catch small insects. Light traps were used to catch nocturnal insects and Malaise traps were used to catch insects that have a tendency to fly upwards.

### Ecological statistical analysis

We used the Dominance Index (DI: McNaughton 1967), Diversity Index (Hʹ: Pielou 1969), Richness Index (RI: Margalef 1973) and Evenness Index (EI: Pielou 1975) as ecological statistical analysis indices:

DI (Dominance Index) = (n1+n2)/N

H' (Diversity Index) = \begin{varwidth}{50in}\begin{equation*}
            -∑(i=1)^s(ni/N ln ni/N)
        \end{equation*}\end{varwidth}

RI (Richness Index) = (S-1)/ln(N)

EI (Evenness Index) = Hʹ/ln(S)

(n1: number of dominant species, n2: number of subdominant species, N: total number of individuals, ni: number of i species, S: total species).

### Cluster analysis

Cluster analysis was performed to examine differences in species composition of invasive alien insects by season, habitat type and region. We used a non-metric multidimensional scaling (NMDS) (McCune and Grace 2002) for this analysis. Seasonal differences were analysed by comparing the abundance of invasive alien insects between April and October. Habitats were categorised into 12 types: farmland, parks, orchards, bare land, roadsides, forests, wetlands, valleys, residential, grassland, waterfront and other sites (schools, airports, city halls and universities). Differences in species richness by region were compared by dividing the eight regions (159 municipalities) into four groups. Group 1 included Jeollanam-do and Jeollabuk-do, Group 2 included Chungcheongnam-do and Chungcheongbuk-do, Group 3 included Gyeongsangnam-do and Gyeongsangbuk-do and Group 4 included Gyeonggi-do and Gangwon-do.

The data values were the number of invasive alien insects species, converted between 0 and 1 and transformed by arcsine square root. Species with fewer than three occurrences were excluded from the analysis to eliminate the effects of small numbers. Distances were calculated using Sørensen (Bray-Curtis) and visually represented using PC-ORD version 7 (McCune and Mefford 1999).

## Results

### National Invasive alien species status

From 2019 to 2021, a total of 95,017 individuals belonging to nine orders, 49 families and 77 species were identified in the nationwide survey of invasive alien insects. Amongst the taxonomic groups, Hemiptera dominated with 27 species (35.1%), followed by Coleoptera with 14 species (18.2%) and Lepidoptera with 11 species (14.3%). The total number of individuals per taxonomic group was 43,146 in the Hemiptera, 14,069 in the Lepidoptera and 13,876 in the Coleoptera (Table 1, Suppl. material 1).

**Figure 2.**
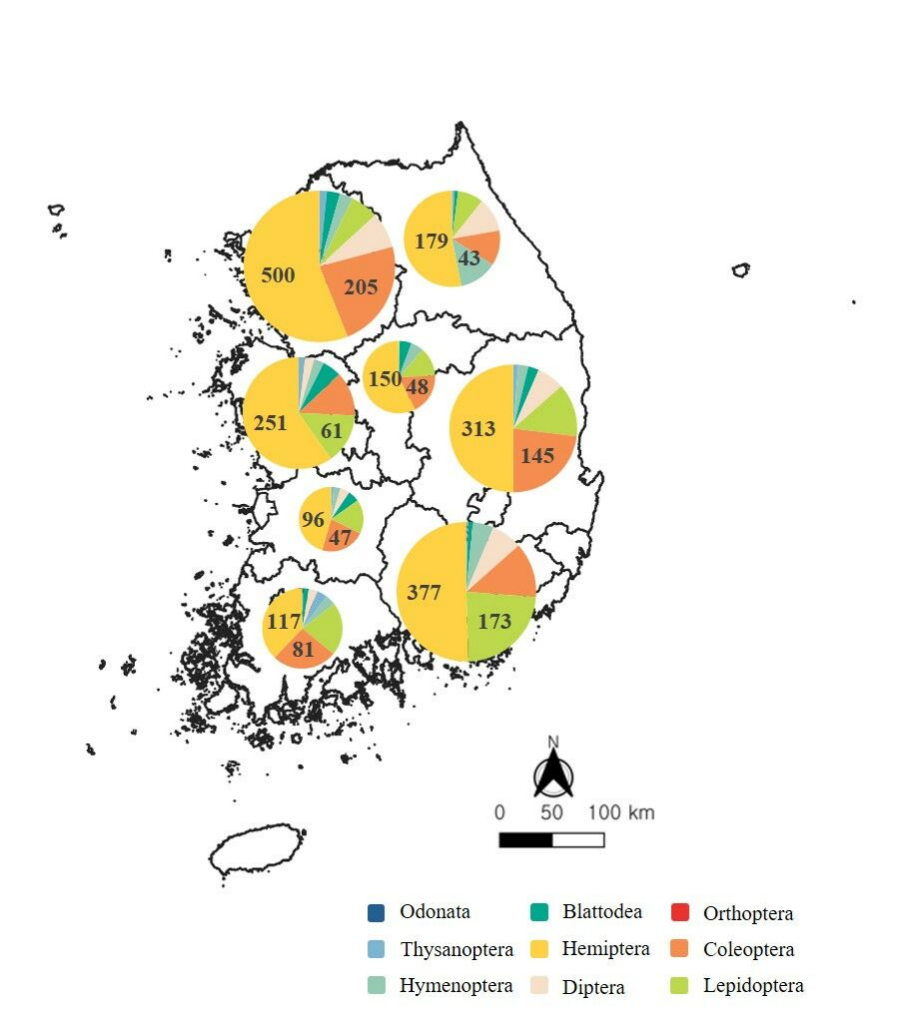
Proportions of taxa according to number of research sites.

The distribution of invasive alien insects according to the survey sites was the highest in Gyeonggi-do, with 890 sites, with Hemiptera and Coleoptera being identified at 500 and 205 sites, respectively. In Gyeongsangnam-do, invasive alien insects were found at 745 sites, with Hemiptera and Lepidoptera being identified at 377 and 173 sites, respectively. In Gyeongsangbuk-do, invasive alien insects were present at 628 sites, with Hemiptera and Coleoptera being identified at 313 and 145 sites, respectively. In Chungcheongnam-do, invasive alien insects were found at 420 sites, with Hemiptera and Lepidoptera being identified at 251 and 61 sites, respectively. In Gangwon-do, invasive alien insects were found at 337 sites, with Hemiptera and Hymenoptera being identified at 179 and 43 sites, respectively. In Jeollanam-do, invasive alien insects were found at 309 sites, with Hemiptera and Coleoptera being identified at 117 and 81 sites, respectively. In Chungcheongbuk-do, invasive alien insects were found at 262 sites, with Hemiptera and Coleoptera being identified at 150 and 48 sites, respectively. In Jeollabuk-do, invasive alien insects were found at 211 sites, with Hemiptera and Coleoptera being identified at 96 and 47 sites, respectively (Fig. 2).

### Invasive alien insect populations by region

In 2019, we surveyed invasive alien insects populations in Jeollanam-do, Jeollabuk-do, Chungcheongnam-do and Chungcheongbuk-do. In Jeollanam-do, we identified a total of 5,482 invasive alien insects belonging to eight orders, 23 families and 28 species. Of these, we identified 10 species from seven families of Hemiptera, followed by five species of Coleoptera and four species of Lepidoptera. Amongst the taxonomic groups, Coleoptera was the most abundant order, with 2,032 individuals, followed by Lepidoptera (1,471 individuals) and Hemiptera (1,104 individuals). In Jeollabuk-do, we identified a total of 3,177 invasive alien insects belonging to seven orders, 20 families and 25 species. Amongst them, Hemiptera was the most abundant order, with nine species in six families, followed by Coleoptera and Lepidoptera, with five species each. The number of individuals per taxonomic group was 1,085 for Hemiptera, 1,014 for Blattodea and 621 for Lepidoptera.

In Chungcheongnam-do, we identified a total of 8,520 invasive alien insects belonging to seven orders, 22 familes and 29 species. Amongst them, Hemiptera was the most abundant order, with eight species in six families, followed by six species of Lepidoptera and five species of Coleoptera. The number of individuals per taxonomic group was 4,157 for Hemiptera, followed by 2,112 Lepidoptera and 1,330 Blattodea. In Chungcheongbuk-do, we identified a total of 8,142 invasive alien insects belonging to six orders, 21 families and 25 species. Amongst them, Hemiptera was the most abundant order, with seven species in five families, followed by six species of Coleoptera and five species of Lepidoptera. The number of individuals per taxonomic group was 5,973 for Hemiptera, 827 for Coleoptera and 594 for Lepidoptera.

In 2020, we surveyed the status of the invasive alien insects populations in Gyeongsangnam-do and Gyeongsangbuk-do. In Gyeongsangnam-do, we identified a total of 23,846 invasive alien insects belonging to eight orders, 41 families and 55 species. Amongst them, Hemiptera was the most abundant order, with 21 species in 15 families, followed by Lepidoptera with nine species and Coleoptera with eight species. The number of individuals per taxonomic group was 8,804 for Hemiptera, 8,487 for Hymenoptera and 2,396 for Lepidoptera. In particular, we confirmed the occurrence of *L.humile* and *M.differentialis*, which have recently been designated ecosystem-disturbing species, in Busan and Ulsan, respectively. In Gyeongsangbuk-do, we also identified a total of 10,649 invasive alien insects belonging to seven orders, 29 families and 40 species. Amongst them, Hemiptera was the most abundant order, with 16 species in 11 families, followed by seven species of Coleoptera and six species of Lepidopera. The number of individuals per taxonomic group was 3,813 for Hemiptera, 2,443 for Blattodea and 2,269 for Coleoptera.

In 2021, we surveyed the status of invasive alien insects populations in Gyeonggi-do and Gangwon-do. In Gyeonggi-do, we identified a total of 18,707 invasive alien insects belonging to seven orders, 28 families and 36 species. Amongst them, Hemiptera was the most abundant order, with 10 species in eight families, followed by Lepidoptera with eight species and Coleoptera with six species. The numbers of individuals per taxonomic group were 8,655 for Hemiptera, 4,652 for Lepidoptera and 3,376 for Coleoptera. Likewise, in Gangwon-do, we identified a total of 16,494 invasive alien insects belonging to seven orders, 29 families and 37 species. Amongst them, Hemiptera was the most abundant order, with 11 species in nine families, followed by Coleoptera with nine species and Lepidoptera with six species. The number of individuals per taxonomic group was 9,555 for Hemiptera, 3,556 for Diptera and 2,253 for Coleoptera (Table 2).

### Ecological statistical analysis

We performed cluster analysis of the invasive alien insects species observed in these eight experimental regions (Table 3). We observed a Dominance Index (DI) ranging from 0.4 to 0.6, with the highest value of 0.6 being detected in Chungcheongbuk-do. Diversity Index (H’) showed a similar pattern, ranging from 2.1 to 2.7, with 2.7 being the highest in Gyeongsangbuk-do and 2.1 being the lowest in Chungcheongbuk-do and Gangwon-do. The Evenness Index (EI) was similar, with values ranging from 0.6 to 0.7 in all provinces. In addition, the Richness Index (RI) ranged from 2.7 to 5.4, with the highest value (5.4) being observed in Gyeongsangnam-do, whereas the lowest value (2.7) was detected in Chungcheongbuk-do.

### Invasive alien species composition by region

**Figure 3.**
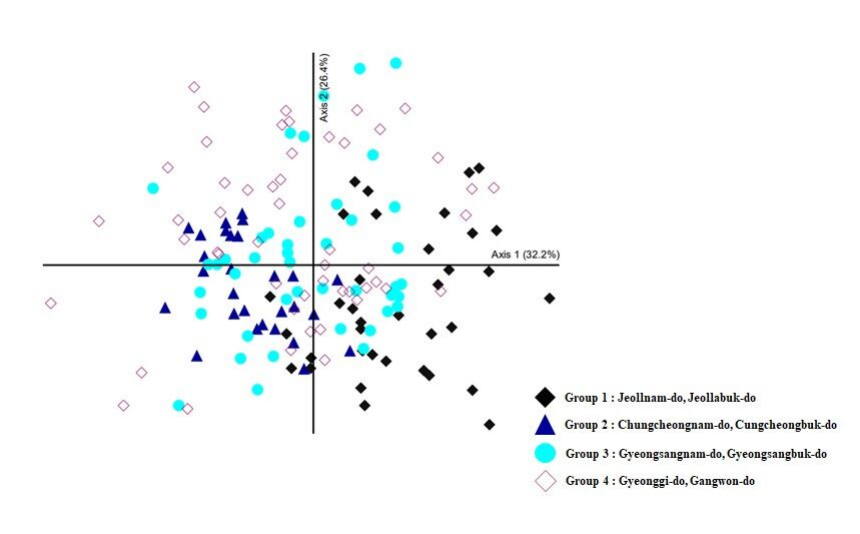
Non-metric Multidimensional Scaling of the four Groups. Axis 1 shows 32.2% of the total variance, while axis 2 shows 26.4%. It was found that there is not a significant difference in the values of Axis 1 and Axis 2.

To examine the differences in the species composition of invasive alien insects across provinces, we analysed the NMDS in the four groups (Fig. 3). We identified 11 species in Group 1, seven species in Group 2, 24 in Group 3 and 11 in Group 4. The invasive alien insects identified in all regions of the country were *Frankliniellaoccidentalis*, *Corythuchaciliate*, *Corythuchamarmorata*, *Leptoglossusoccidentalis*, *Ricaniasublimata*, *Metcalfapruinosa*, Lycorma delicauta (all Hemiptera), *Ophraellacommuna*, *Lissorhoptrusoryzophilus*, *Hyperapostica*, *Ceutorhynchusobstructus* (all Coleoptera), *Vespavelutinanigrithorax*, *Bombusterrestris* (all Hymenoptera), *Hyphantriacunea* (Lepidoptera) and others. The invasive alien insects found in Groups 3 and 4 overlapped extensively.

### Differences in species composition over seasonal changes

**Figure 4.**
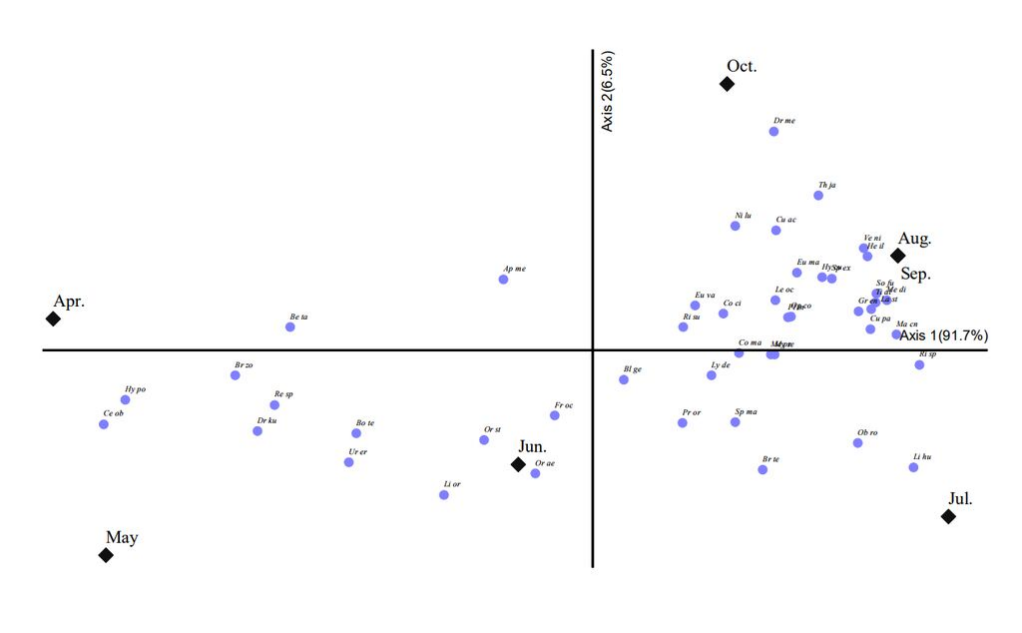
Non-metric Multidimensional Scaling of the seasonal species composition analysis. Axis 1 shows 91.7% of the total variance, while axis 2 shows 6.5%. Abbreviations Blge: *Blattellagermanica*, Resp: *Reticulitermessperatuskyushuensis*, Medi: *Melanoplusdifferentialis*, Froc: *Frankliniellaoccidentalis*, Orae: *Oriusaevigatuslaevigatus*, Orst: *Oriusstrigicollis*, Coci: *Corythuchaciliate*, Coma: *Corythuchamarmorata*, Leoc: *Leptoglossusoccidentalis*, Risu: *Ricaniasublimate*, Mepr: *Metcalfapruinosa*, Lyde: *Lycormadelicatula*, Nilu: *Nilaparvatalugens*, Last: *Laodelphaxstriatellus*, Sofu: *Sogatellafurcifera*, Urer: *Uroleuconerigeronense*, Beta: *Bemisiatabaci*, Opco: *Ophraellacommuna*, Lior: *Lissorhoptrusoryzophilus*, Hypo: *Hyperapostica*, Brzo: *Brachyperazoilus*, Drku: *Dryocosmuskuriphilus*, Veni: *Vespavelutinanigrithorax*, Apme: *Apismellifera*, Bote: *Bombusterrestris*, Tial: *Tineariaalternate*, Obro: *Obolodiplosisrobiniae*, Thja: *Thecodiplosisjaponensis*, Heil: *Hermetiaillucens*, Gren: *Grapholitaendrosias*, Euva: *Eumetavariegate*, Hycu: *Hyphantriacunea*, Plin: *Plodiainterpunctella*, Macn: *Macrocentruscnaphalocrocis*, Myse: *Mythimnaseparata*, Spex: *Spodopteraexigua*, Euma: *Euremamandarina*, Cuac: *Curetisacuta*.

We then analysed the species composition by month to determine whether species composition differed with seasonal changes (Fig. 4). We identified 26 species in April, 33 species in May, 36 species in June, 53 species in July, 45 species in August, 41 species in September and 36 species in October. We observed differences in the number of invasive alien insects identified across seasons. Between April and October, the most common species identified were *Blattellagermanica* (Blattodea), *Frankliniellaoccidentalis*, *Corythuchaciliate*, *Corythuchamarmorata*, *Leptoglossusoccidentalis*, *Ricaniasublimate*, *Metcalfapruinosa*, *Lycormadelicatula* (all Hemiptera), *Ophraellacommuna* (Coleoptera) and *Euremamandarina* (Lepidoptera). In spring and summer, the species identified included *Oriusaevigatuslaevigatus* (Hemiptera), *Lissorhoptrusoryzophilus*, *Hyperapostica*, *Brachyperazoilus* (all Coleoptera) and *Dryocosmuskuriphilus* (Hymenoptera). Whereas, after summer, the species identified included *Vespavelutinanigrithorax* (Hymenoptera), *Thecodiplosisjaponensis*, *Hermetiaillucens* (all Diptera), *Laodelphaxstriatellus*, *Sogatellafurcifera* (all Hemiptera), *Macrocentruscnaphalocrocis*, *Grapholitaendrosias*, *Plodiainterpunctella*, *Mythimnaseparata* (all Lepidoptera) and others.

### Habitat environment type

**Figure 5.**
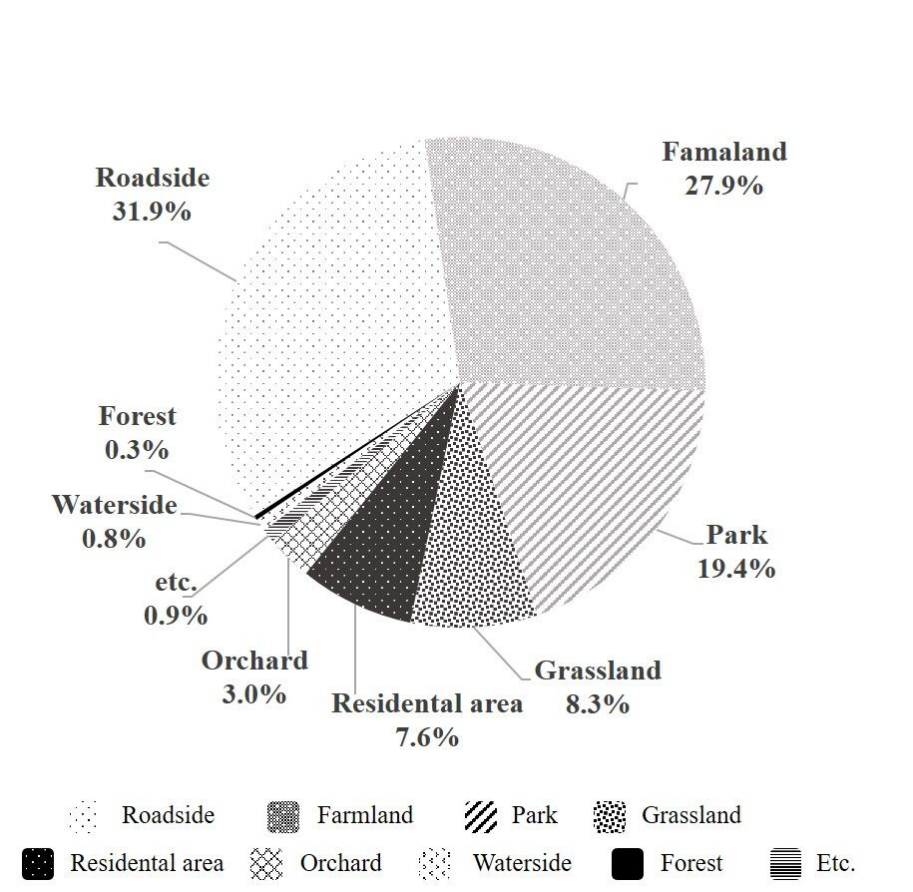
Types of habitats used by invasive alien insects in South Korea.

**Figure 6.**
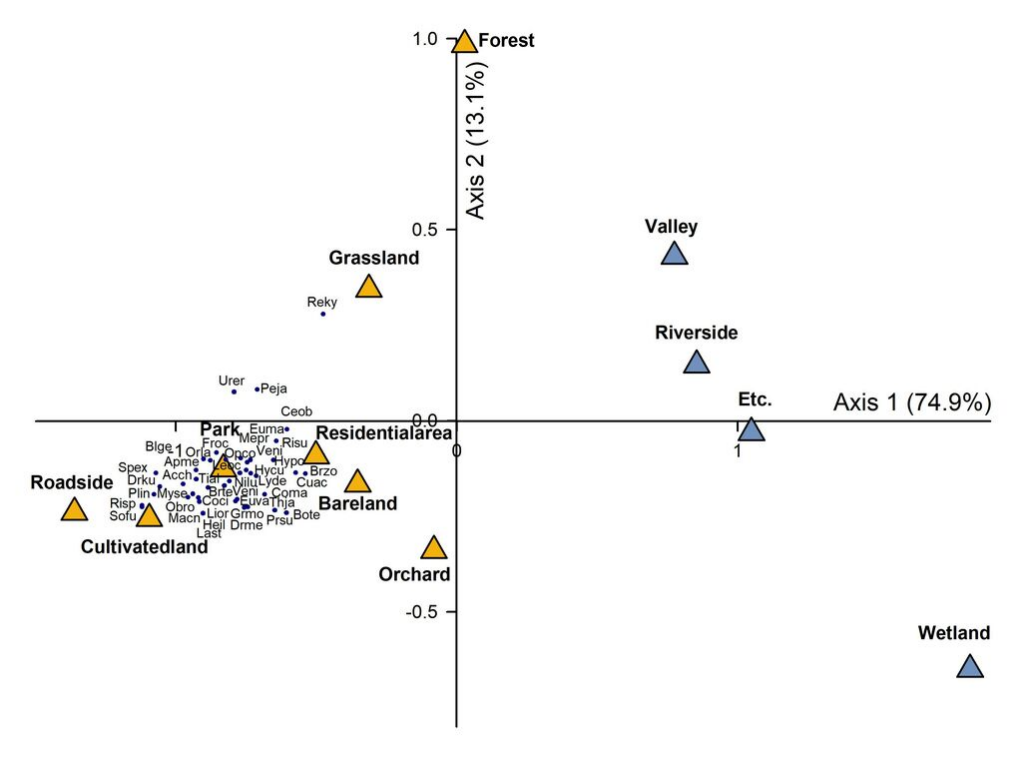
Non-metric Multidimensional Scaling of the habitat environment types. Axis 1 shows 74.9% of the total variance, while axis 2 shows 13.1%. Habitat types preferred by invasive alien insects, with yellow triangles representing dried and disturbed areas and blue triangles representing waterfront or natural areas (excluding etc.). Abbreviations Acch: *Acyrthosiphonchelidonii*, Apme: *Apismellifera*, Blge: *Blattellagermanica*, Bote: *Bombusterrestris*, Brte: *Brachymnatenuis*, Brzo: *Brachyperazoilus*, Ceob: *Ceutorhynchusobstructus*, Coci: *Corythuchaciliate*, Coma: *Corythuchamarmorata*, Cuac: *Curetisacuta*, Drku: *Dryocosmuskuriphilus*, Drme: *Drosophilamelanogaster*, Euma: *Euremamandarina*, Euva: *Eumetavariegate*, Froc: *Frankliniellaoccidentalis*, Grmo: *Grapholitamolesta*, Heil: *Hermetiaillucens*, Hycu: *Hyphantriacunea*, Hypo: *Hyperapostica*, Last: *Laodelphaxstriatellus*, Leoc: *Leptoglossusoccidentalis*, Lior: *Lissorhoptrusoryzophilus*, Lyde: *Lycormadelicatula*, Macn: *Macrocentruscnaphalocrocis*, Mepr: *Metcalfapruinosa*, Myse: *Mythimnaseparata*, Nilu: *Nilaparvatalugens*, Obro: *Obolodiplosisrobiniae*, Opco: *Ophraellacommuna LeSage*, Orla: *Oriuslaevigatuslaevigatus*, Peja: *Periplanetajaponica*, Plin: *Plodiainterpunctella*, Prsu: *Protaetiaorientalissubmarmorea*, Reky: *Reticulitermessperatuskyushuensis*, Risp: *Ricaniaspeculum*, *Risu*: *Ricaniasublimata*, Sofu: *Sogatellafurcifera*, Spex: *Spodopteraexigua*, Thja: *Thecodiplosisjaponensis*, Tial: *Tineariaalternata*, Urer:*Uroleuconerigeronense*, Veni: *Vespavelutinanigrithorax*.

We also analysed the habitats to determine the habitat environments where invasive alien insects were found. The most common habitats of invasive alien insects were roadsides, farmlands, parks, residences, grasslands, orchards, wetlands, valleys, streams and forests. In particular, roadside was the most common habitat (31.9 %), followed by croplands (27.9 %) and parks (19.4 %) (Fig. 5). When analysing habitat types using NMDS, we found two main groups were formed along the first axis. The first group consisted of roadsides, grasslands, parks, bare land, orchards, cultivated fields, residences and forests. The second group consisted of valleys, waterfronts, wetlands and other environments. The first group was the dominant and preferred environment for invasive alien insects, with higher densities in dry and disturbed environments (Fig. 6).

## Discussion

In this study, we analysed the current status of invasive alien insects found in natural ecosystems. In particular, we identified a total of 77 species from 49 families and nine orders of invasive alien insects in the nationwide survey. Previously, a total of 63 invasive alien insects belonging to 43 families and nine orders were identified from 2015 to 2018 (Kim et al. 2022); however, a greater number of invasive alien insects were identified in this study. As 26 species were newly identified in this study, we expect that the number of invasive alien insects found in natural ecosystems will continue to increase. Once introduced into the country through ports, such as the *L.humile* and *M.differentialis*, they are likely to spread throughout the country because they can spread long distances by rail and other modes of transportation and are highly adaptable to new environments (Kiritani and Morimoto 2004, Hulme et al. 2008).

The most important factors affecting the range expansion of invasive alien insects are their reproductive rates and dispersal abilities (Kiritani and Morimoto 2004). In particular, true bugs and moths are likely to be introduced in the form of egg masses by laying eggs on the surfaces of ships, containers, and cargo (Dara et al. 2015, Paini et al. 2018), which are difficult to detect, making them relatively easy to introduce and establish (Meurisse et al. 2019). Invasive alien insects have been reported to spread faster than native species, with Hemiptera being able to spread 66.9 km per year and multivoltine insects moving 56 km per year faster on average than univoltine insects (Fahrner and Aukema 2018). In this study, Hemiptera were found in all regions of the country, with 43,146 individuals at 1,983 habitats. The reasons for the high number of Hemiptera were that invasive alien species are known to spread faster than native species (Roques et al. 2016) and factors such as the availability of host, envirenmental factors, the number of eggs laid at a time and the number of repeated generations are likely to be significant (Liang et al. 2016, Jung et al. 2022, Kalaitzaki et al. 2023). Due to various factors, it is speculated that there are many Hemiptera invasive alien insects found in South Korea. In addition to natural spread, anthropogenic transport through highways and railroads can facilitate the spread and establishment of invasive alien insects, giving them the advantage of spreading to more areas (Kiritani and Morimoto 2004, Prasad et al. 2010).

Invasive alien insects have been identified throughout the country. To determine whether the species composition of invasive alien insects spread across the country differed across regions, we used various methods to compare and analyse their composition by region. A greater number of invasive alien insects were found in Gyeongsangbuk-do and Gyeonggi-do than in Jeolla-do and Chungcheongbuk-do, with a higher number of habitats in these regions. According to Cadotte et al. (2017), significant urbanisation or human disturbance in the habitat increases the accessibility of invasive alien insects resulting in conditions that are favourable for their establishment. Gyeonggi-do has a high urbanisation index compared with other regions because of the increase in population, as Seoul, Incheon and other surrounding satellite cities are in this region (Kim 2010). Consequently, it is likely to be more ecologically disturbed compared to other cities and may include more habitat sites for invasive alien insects because the environment in which invasive alien insects live may be more diverse than that in other provinces. On the one hand, the invasive alien insects identified in Gyeongsang-do were more diverse compared with those in other regions. The reason for the large number of invasive alien insects species is attributed to the many major ports and cargo terminals, from where invasive alien insects are easily introduced (Koch et al. 2011). According to Lee et al. (2021), of the 10 major ports in South Korea, three are located in Gyeongsang-do; the Busan, Ulsan and Pohang Ports. Amongst them, 74.5% of containers are imported through Busan Port, which has the highest share of container imports amongst the major ports in South Korea. Invasive alien insects can easily settle in areas close to cargo terminals and they are likely to spread to surrounding areas through containers or other means of transportation; therefore, the number of invasive alien insects species in this region was higher than that in other regions.

The main habitats in which invasive alien insects were found were roadsides, croplands, parks, residences and orchards. More specifically, 31.9% of invasive alien insects were found along roadsides. The ease of observation of invasive alien insects along roadsides is likely to be influenced by the type of road and mode of transportation. Lemke et al. (2019) reported that ragweed (*Ambrosiaartemisiifolia*) is more likely to spread by transportation than by natural spread, while Yemshanov et al. (2012) reported that insects can escape from cargo vehicles in the course of trade and move to highways or forests. In addition to roadsides, invasive alien insects were also found in parks and agricultural fields. Flower beds with woody or herbaceous plants and surrounding plants that are available as food sources are likely to be the main habitats for invasive alien insects as they maintain ecological diversity and provide suitable environments for insects, such as shelter (New et al. 2021). When analysing habitat types using NMDS, the distribution of invasive alien insects was concentrated in arid and anthropogenic environments, with a higher preference in anthropogenic environments, such as roadsides, parks, residences, croplands, orchards, grasslands and bare land. As they become established and adapt to disturbed urban environments rather than stable natural habitats, the number of species available for colonisation and their populations are expected to increase in the future (Marques et al. 2020).

In disturbed urban environments, invasive alien insects become more adaptable (Branco et al. 2019) and increase in size, reproductive rate and density (Dale and Frank 2017). Increased impervious surfaces, such as roads and buildings and warmer temperatures, have been associated with an increase in the density of street tree pests and urban invasive alien insects (Adams et al. 2020). Increasing temperatures directly affect insect population regulation, including insect survival, developmental rate, fecundity and dispersal (Bale et al. 2002). Of note, increasing temperatures play an important role in increasing native species populations and facilitating the establishment and spread of invasive alien insects (Robinet and Roques 2010). Of the 77 invasive alien insects identified in this study, 53 species were identified in July and over 40 species were identified in August and September. Warmer temperatures are likely to have led to an increase in the numbers of invasive alien insects by diversifying the host plants they utilise as food sources (Ju et al. 2017). Their fecundity is dependent on the summer photoperiod and suitable temperatures and continued increases in climate temperatures due to global warming could result in an increase in the number of generations per year and an expansion of their distribution range (Bale et al. 2002, Kiritani and Morimoto 2004, Laštůvka 2009). Moreover, warmer temperatures may have accelerated flowering and pushed back the insect hatching season (Ju et al. 2017), which may explain the higher abundance of invasive alien insects species in summer than in other seasons. However, further research is required to determine the causal relationship between rising temperatures and the increase in the numbers of invasive alien insects. Warmer winter temperatures are expected to increase the number of invasive alien insects that are capable of overwintering and that of insects that become active in early spring, increasing the likelihood of large outbreaks (Bale et al. 2002).

In this study, we investigated the status of invasive alien insects introduced and settled in natural ecosystems in South Korea. Despite strengthening the quarantine process in border areas to manage the introduction of invasive alien insects into South Korea (Hong et al. 2012, Lee et al. 2016, Kang et al. 2020), the number of invasive alien insects introduced into South Korea continues to increase. Estimating the timing and status of the introduction of invasive alien insects through the blind spots of quarantine and prevention is difficult, making efficient management also difficult (Kiritani and Morimoto 2004). Therefore, understanding the status of invasive alien insects populations, their preferred habitats, seasonal characteristics, differences in species composition by region and ecological characteristics is necessary for the efficient management of invasive alien insects. Invasive alien insects are characterised by their environmental adaptability, rapid spread, high fecundity and host diversity (Mainka and Howard 2010). As their spread is relatively rapid compared with that of native insects (Fahrner and Aukema 2018), they have the potential to spread across the country in a short period of time and research is needed to understand their migration routes, timing and geographic impacts. Preventing the introduction of invasive alien insects in advance is the most effective method; however, this is difficult because of factors such as increasing international trade, human migration and climate change. Therefore, conducting habitat surveys of already introduced invasive alien insects is necessary for the early identification and prediction of their potential spread in the ecosystem, as well as management to minimise damage to the ecosystem. Continuously monitoring is necessary to minimise the impact of invasive insects on biodiversity, agriculture and public health.

## Supplementary Material

92E3F3B4-787B-5EE9-8147-96C174F90839 10.3897/BDJ.12.e133668.suppl1 Supplementary material 1 List of invasive alien insects identified in South Korea Data type List of invasive alien insects. File: oo_1131955.xlsx https://binary.pensoft.net/file/1131955 National Institute of Ecology

## Figures and Tables

**Table 1.** Status of invasive alien insects by taxon.

Order	Family	Species	% of species	No. of individuals	% of individuals	Research sites
Odonata	1	1	1.3	12	0.0	1
Blattodea	3	5	6.5	6,849	7.2	110
Orthoptera	1	1	1.3	700	0.7	1
Thysanoptera	1	4	5.2	1,191	1.3	52
Hemiptera	16	27	35.1	43,146	45.4	1,983
Coleoptera	9	14	18.2	13,876	14.6	715
Hymenoptera	4	7	9.1	9,745	10.3	167
Diptera	5	7	9.1	5,429	5.7	236
Lepidoptera	9	11	14.3	14,069	14.8	537
Total	49	77	100.0	95,017	100.0	3,802

**Table 2.** Status of invasive alien insects by region.

Jeollanam-do					Jeollabuk-do				
Order	Family	Species	Research sites	No. of individuals	Order	Family	Species	Research sites	No. of individuals
Odonata	1	1	1	12					
Blattodea	2	2	7	686	Blattodea	1	1	12	1,014
Thysanoptera	1	1	13	121	Thysanoptera	1	1	3	18
Hemiptera	7	10	117	1,104	Hemiptera	6	9	96	1,085
Coleoptera	3	5	81	2,032	Coleoptera	3	5	47	353
Hymenoptera	3	3	13	26	Hymenoptera	2	2	7	10
Diptera	2	2	11	30	Diptera	2	2	10	76
Lepidoptera	4	4	66	1,471	Lepidoptera	5	5	36	621
Total	23	28	309	5,482	Total	20	25	211	3,177
Chungcheongnam-do					Chungcheongbuk-do				
Order	Family	Species	Research sites	No. of individuals	Order	Family	Species	Research sites	No. of individuals
Blattodea	3	3	23	1,330	Blattodea	3	3	14	231
Thysanoptera	1	3	8	16	Thysanoptera	1	1	1	2
Hemiptera	6	8	251	4,157	Hemiptera	5	7	150	5,973
Coleoptera	3	5	54	820	Coleoptera	4	6	48	827
Hymenoptera	2	2	12	49	Hymenoptera	3	3	15	515
Diptera	2	2	11	36					
Lepidoptera	5	6	61	2,112	Lepidoptera	5	5	34	594
Total	22	29	420	8,520	Total	21	25	262	8,142
Gyeongsangnam-do					Gyeongsangbuk-do				
Order	Family	Species	Research sites	No. of individuals	Order	Family	Species	Research sites	No. of individuals
Blattodea	3	3	8	73	Blattodea	3	3	17	2,443
Orthoptera	1	1	1	700					
Thysanoptera	1	2	2	671	Thysanoptera	1	1	8	185
Hemiptera	15	21	377	8,804	Hemiptera	10	16	313	3,813
Coleoptera	5	8	95	1,946	Coleoptera	4	7	145	2,269
Hymenoptera	4	5	36	8,487	Hymenoptera	2	2	16	56
Diptera	5	6	53	769	Diptera	3	4	44	438
Lepidoptera	7	9	173	2,396	Lepidoptera	5	6	85	1,445
Total	41	55	745	23,846	Total	29	39	628	10,649
Gyeonggi-do					Gangwon-do				
Order	Family	Species	Research sites	No. of individuals	Order	Family	Species	Research sites	No. of individuals
Blattodea	3	4	25	1,058	Blattodea	2	2	4	14
Thysanoptera	1	1	14	144	Thysanoptera	1	2	3	34
Hemiptera	8	10	500	8,655	Hemiptera	9	11	179	9,555
Coleoptera	3	6	205	3,376	Coleoptera	7	9	40	2,253
Hymenoptera	3	3	25	298	Hymenoptera	3	4	43	304
Diptera	4	4	68	524	Diptera	2	3	39	3,556
Lepidoptera	6	8	53	4,652	Lepidoptera	5	6	29	778
Total	28	36	890	18,707	Total	29	37	337	16,494

**Table 3.** Ecological statistical analysis for eight regions.

Research areas	Dominance Index (DI)	Diversity Index (H')	Evenness Index (EI)	Richness Index (RI)
Jeollanam-do	0.4	2.3	0.7	3.1
Jeollabuk-do	0.5	2.2	0.7	3.0
Chungcheongnam-do	0.4	2.3	0.7	3.1
Chungcheongbuk-do	0.6	2.1	0.6	2.7
Gyeongsangnam-do	0.5	2.5	0.6	5.4
Gyeongsanbuk-do	0.4	2.7	0.7	4.2
Gyeonggi-do	0.4	2.4	0.7	3.6
Gangwon-do	0.4	2.1	0.6	3.7
